# Identification of serum sirtuins as novel noninvasive protein markers for frailty

**DOI:** 10.1111/acel.12260

**Published:** 2014-08-07

**Authors:** Rahul Kumar, Navinath Mohan, Ashish Datt Upadhyay, Amrendra Pratap Singh, Vishal Sahu, Sadanand Dwivedi, Aparajit B Dey, Sharmistha Dey

**Affiliations:** 1 Department of Biophysics, All India Institute of Medical SciencesNew Delhi, 110029, India; 2 Department of Geriatric Medicine, All India Institute of Medical SciencesNew Delhi, 110029, India; 3 Department of Biostatistics, All India Institute of Medical SciencesNew Delhi, 110029, India

**Keywords:** biomarker, frailty, serum, sirtuin, surface plasmon resonance

## Abstract

Frailty has emerged as a major health issue among older patients. A consensus on definition and diagnosis is yet to be achieved. Various biochemical abnormalities have been reported in frailty. Activation of sirtuins, a conserved family of NAD-dependent proteins, is one of the many mimics of calorie restriction which improves lifespan and health in experimental animals. In this cross-sectional study, we assessed the circulating sirtuin levels in 119 (59.5%) nonfrail and 81 (40.5%) frail individuals, diagnosed by Fried's criteria. Serum SIRT1, SIRT2, and SIRT3 were estimated by surface plasmon resonance (SPR) and Western blot. Serum sirtuins level in mean+SD; SIRT1 (nonfrail –4.67 ± 0.48 ng/μL; frail – 3.72 ± 0.48 ng/μL; *P* < 0.0001), SIRT2 (nonfrail – 15.18 ± 2.94 ng/μL; frail – 14.19 ± 2.66 ng/μL; *P* = 0.016), and SIRT3 (nonfrail-7.72 ± 1.84 ng/μL; frail – 6.12 ± 0.97 ng/μL; *P* < 0.0001) levels were significantly lower among frail patients compared with the nonfrail. In multivariable regression analysis, lower sirtuins level were significantly associated with frailty after adjusting age, gender, diabetes mellitus, hypertension, cognitive status (Mini Mental State Examination scores) and number of comorbidities. For detecting the optimum diagnostic cutoff value a ROC analysis was carried out. The area under curve for SIRT1 was 0.9037 (cutoff – 4.29 ng/μL; sensitivity – 81.48%; specificity – 79.83%) and SIRT3 was 0.7988 (cutoff – 6.61 ng/μL; sensitivity – 70.37%; specificity – 70.59%). This study shows that lower circulating SIRT1 and SIRT3 levels can be distinctive marker of frailty.

## Introduction

Frailty is a complex clinical state in old age. There is no consensus regarding its definition and diagnosis (Rockwood, 2005). Manifestations of frailty include sarcopenia (loss of skeletal muscle mass); cognitive decline; abnormal functioning of immune and neuroendocrine systems; and poor energy regulation (Clegg *et al*., 2013). It is a state of multi-organ dysfunction at cellular and subcellular level leading to decreased physiologic reserve, increased vulnerability to stressors, serious adverse health outcomes (Lipsitz & Goldberger, 1992; Bortz, 1993; Fried *et al*., 2001). Aging or senescence is a strong risk factor for developing frailty with accumulation of senescent cells in tissues and organs over the lifetime (Heemels, 2010). Cytokines released during pathogenesis of various age-related diseases also increase the risk of frailty by lipolysis, muscle protein breakdown, and nitrogen loss (Bales & Ritchie, 2002).

Diagnosis of frailty is often difficult because of subtle and subjective clinical features, especially in the early stage of the syndrome. There is no definitive treatment for frailty, which implies that the conditions needs to be detected early and intervened to arrest further progression to dependence. An objective marker for early diagnosis of frailty remains elusive till date. Considering the strong association of frailty with senescence, it is essential to explore molecules linked to the process of senescence. Sirtuin proteins (silent information regulator) have emerged as a epigenetic regulator of senescence in yeast (Kaeberlein *et al*., 1999), *Caenorhabditis elegans (*Tissenbaum & Guarente, 2001), *Drosophila melanogaster* (Rogina & Helfand, 2004) and mice (Herranz *et al*., 2010). Sirtuins belong to a family of nicotinamide adenine dinucleotide (NAD)-dependent protein deacetylases (Imai *et al*., 2000). The anti-aging properties of sirtuins activation are possibly related to key reactions in biological effects of calorie restriction. Activation of sirtuins improves lifespan and health in experimental animals (Mitchell *et al*., 2014). There are not many reports on sirtuins in human aging. We have reported significantly lower circulating sirtuin levels in older subjects compared with the healthy young adults (Kumar *et al*., 2013).

One of the most potential approaches to identify protein markers for a disease is by analyzing human body fluid (e.g. blood, urine, saliva) proteome. Human serum proteins originate from different tissues and enter the circulation as a result of secretion and leakage (Taylor, 1969). The concentration of these proteins reflects human physiological or pathological state as suggested by several earlier reports (Anderson & Anderson, 2002; Thadikkaran *et al*., 2005). As frailty is not any tissue- or organ-specific condition, we considered serum samples as ideal source for biomarkers. In this cross-sectional study, association of serum sirtuin concentration was assessed in frail and nonfrail older subjects with an objective of examining it as a marker of frailty in old age.

## Results

### Baseline data

Two hundred patients (≥60 years of age) attending Geriatric Medicine Outpatient Department for various indications were assessed for frailty status using Fried's criteria. Of them, 119 (59.5%) were classified as nonfrail and 81 (40.5%) as frail. Table1 provides baseline demographic characteristics of the subjects. Mean age of frail patients was significantly higher (*P *< 0.0001) than nonfrail subjects. There was no significant difference in gender (*P *=* *0.378) and nutritional status [BMI (*P *=* *0.102)] between the two groups. Diabetes mellitus (34%) and hypertension (32%) were common comorbidities. The prevalence of diabetes (*P *< 0.0001) and hypertension (*P *=* *0.009) was significantly higher among the frail subjects. There was no significant difference in prevalence of other comorbidities.

**Table 1:** Baseline data of frail and nonfrail subjects

*N*	Nonfrail 119	Frail 81	*P*-value
Age (years), mean ± SD	68.41 ± 3.55	74.34 ± 6.59	<0.0001
Male, *n* (%)	69 (57.98)	52 (64.2)	0.378
BMI, mean ± SD	23.78 ± 3.04	22.96 ± 4.08	0.102
MMSE, mean ± SD	25.94 ± 2.25	24.95 ± 2.87	0.007
Diabetes Mellitus, *n* (%)	28 (23.53)	40 (49.38)	<0.0001
COAD, *n* (%)	12 (10.08)	14 (17.28)	0.137

### Estimated Sirtuin level

#### By surface plasmon resonance

**Figure 1:**
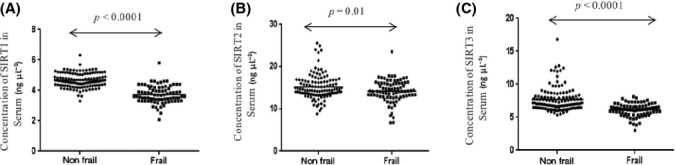
Scatter graph showing the concentration of serum SIRT1(A), SIRT2(B), and SIRT3(C) in frail and nonfrail subjects.

The response unit (RU) of immobilized antibodies; SIRT1 SIRT2, and SIRT3 were 4468, 2473, and 5426, respectively, where 1 RU corresponding to 1 pg/mm^2^. A standard curve was plotted with RU obtained from the sensogram with different concentrations of pure SIRT1 SIRT2, and SIRT3. The binding of the ligands, that is, sirtuins, was in the linear range which is shown in the Fig. S1 (A–C) (Supporting information). Levels of SIRT1 SIRT2, and SIRT3 were significantly lower in frail subjects as compared to the nonfrail as shown in Fig1. After adjustment for age, sex, diabetes mellitus, hypertension, cognitive status (MMSE scores), and number of co morbidities, serum SIRT1, SIRT2, and SIRT3 concentrations were found to be significantly lower in frail as compared to nonfrail patients (Table2). Irrespective of various categories such as differences in age (>60), sex, diabetes mellitus, hypertension, and number of comorbidities, the SIRT1 (Table3) and SIRT3 (Table4) levels were still significantly lower in case of frail as compared to nonfrail; however, in case of SIRT2, the differences were not significant (Table5).

**Table 2:** Unadjusted and adjusted values (ng/μL) represented as mean ± SE

Protein	Unadjusted/Adjusted	Nonfrail	Frail	*P*-value
SIRT1	Unadjusted	4.67 ± 0.04	3.72 ± 0.63	<0.0001
	Adjusted	4.68 ± 0.05	3.71 ± 0.07	<0.0001
SIRT2	Unadjusted	15.19 ± 0.27	14.19 ± 0.29	<0.01
	Adjusted	15.20 ± 0.28	14.17 ± 0.36	0.047
SIRT3	Unadjusted	7.71 ± 0.17	6.12 ± 0.11	<0.0001
	Adjusted	7.7 ± 0.15	6.14 ± 0.19	<0.0001

**Table 3:** Concentration of SIRT1(ng/μL) represented as mean ± SD with respect to different characteristics

	Nonfrail	Frail	*P*-value
Age			
60–69	4.69 ± 0.5	3.7 ± 0.61	<0.0001
70–79	4.6 ± 0.43	3.69 ± 0.53	<0.0001
≥80	5.12 ± 0.11	3.84 ± 0.67	0.02
Gender			
Male	4.64 ± 0.40	3.77 ± 0.59	<0.0001
Female	4.7 ± 0.56	3.64 ± 0.53	<0.0001
Diabetes			
Yes	4.65 ± 0.58	3.69 ± 0.49	<0.0001
No	4.67 ± 0.44	3.76 ± 0.64	<0.0001
Hypertension			
Yes	4.73 ± 0.53	3.68 ± 0.57	<0.0001
No	4.64 ± 0.46	3.76 ± 0.58	<0.0001
No. of comorbidities			
0 or 1	4.69 ± 0.45	3.77 ± 0.67	<0.001
2	4.56 ± 0.60	3.77 ± 0.51	<0.001
≥3	4.77 ± 0.52	3.51 ± 0.46	0.001

**Table 4:** Concentration of SIRT3(ng/μL) represented as mean ± SD with respect to different characteristics

	Nonfrail	Frail	*P-*value
Age			
60–69	7.67 ± 1.96	5.93 ± 0.96	0.001
70–79	7.76 ± 1.6	6.11 ± 1.04	<0.0001
≥80	8.46 ± 2.72	6.3 ± 0.79	0.01
Gender			
Male	7.72 ± 1.84	6.18 ± 1.01	<0.0001
Female	7.7 ± 1.86	6.0 ± 0.91	<0.0001
Diabetes			
Yes	7.5 ± 1.48	5.9 ± 0.97	<0.0001
No	7.78 ± 1.94	6.53 ± 0.93	<0.0001
Hypertension			
Yes	7.37 ± 1.5	6.09 ± 0.86	<0.0001
No	6.13 ± 1.06	7.81 ± 1.92	<0.0001
No. of comorbidities			
0 or 1	7.81 ± 1.91	6.24 ± 1.24	<0.0001
2	7.25 ± 1.50	6.09 ± 0.83	0.001
≥3	7.74 ± 1.77	5.93 ± 0.63	0.00

**Table 5:** Concentration of SIRT2(ng/μL) represented as mean ± sd with respect to different characteristics

	Nonfrail	Frail	*P*-value
Age			
60–69	15.16 ± 3.03	13.64 ± 3.06	0.07
70–79	15.27 ± 2.88	14.21 ± 2.75	0.08
≥80	14.39 ± 1.16	14.62 ± 2.03	0.88
Gender			
Male	15.25 ± 2.70	14.24 ± 2.4	0.03
Female	15.09 ± 3.27	14.11 ± 3.13	0.19
Diabetes			
Yes	15.44 ± 2.07	14.20 ± 2.52	0.04
No	15.10 ± 3.17	14.18 ± 2.82	0.11
Hypertension			
Yes	14.94 ± 2.87	13.82 ± 2.26	0.09
No	13.55 ± 2.87	15.58 ± 3.01	0.0001
No. of comorbidities			
0 or 1	15.21 ± 3.17	13.54 ± 3.24	0.01
2	15.15 ± 1.46	14.53 ± 2.24	0.2
≥3	14.43 ± 4.02	14.76 ± 2.08	0.82

**Figure 2:**
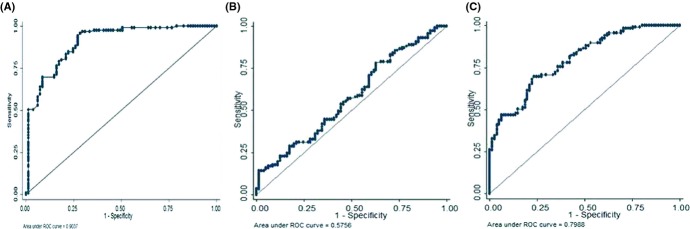
ROC analysis showing the area under curve for SIRT1(A), SIRT2(B), and SIRT3(C) to distinguish frail from nonfrail subjects.

Receiver operating characteristic (ROC) curves were generated for each protein. Based on the Surface Plasmon Resonance (SPR) data, the area under the ROC curves was calculated to measure the utility of each protein as potential marker for frailty. ROC curves are traditionally constructed for detecting disease having higher values of concerned marker. However, in our study, lower levels were associated with disease condition; hence, the ROC curves were constructed to detect nonfrails. The calculated area for predicting nonfrails was 0.9037 for SIRT1, 0.5756 for SIRT2, and 0.7988 for SIRT3 (Fig.2), suggesting that both SIRT1 and SIRT3 can detect nonfrail. Threshold for detecting frailty was chosen based on the distribution of specificities and sensitivities. Based on our data, for SIRT1, a threshold value of ≥4.29 ng/μL yields a sensitivity of 79.83% and a specificity of 81.48% to detect nonfrails or else it can be stated that at a cutoff value of <4.29 ng/μL can detect frailty with sensitivity of 81.48% and specificity of 79.83%. Similarly, for SIRT3, a threshold value of ≥6.61 ng/μL yields a sensitivity of 70.59% and specificity of 70.37% to detect nonfrail individuals, or at cutoff value <6.61 ng/μL, SIRT3 has a sensitivity of 70.37% and specificity of 70.59% to detect frailty. Cutoff value for SIRT2 was not calculated as the area under curve was very low. However, the diagnostic efficiency of SIRT1 to detect frailty was higher as compared to SIRT3.

#### By Western blot

**Figure 3:**
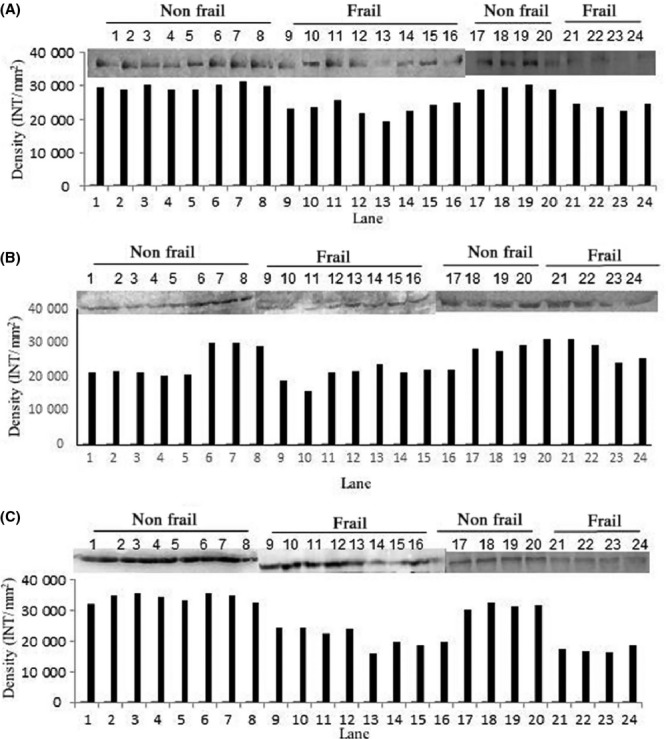
Western blot and density analysis to confirm the presence of SIRT1 (A), SIRT2(B), and SIRT3(C) in serum of nonfrail and frail subjects.

Western blot analysis of the serum samples was used to validate differential expression of SIRT1, SIRT2, and SIRT3 in frails and nonfrails obtained by SPR data. These results are clearly consistent with the lower band density of SIRT1, SIRT2, and SIRT3 in frail when compared to nonfrail (Fig3A–C) as demonstrated by SPR data. The linearity of the sensitivity and specificity of different antibodies was illustrated in Fig S2 (A–C) (Supporting information).

## Discussion

Sirtuins has generated considerable interest as an important player in aging biology. It is generally agreed that sirtuins have a role in expanding the lifespan in laboratory model organisms by mediating the anti-aging effects of a low-calorie diet (calorie restriction) (Haigis & Guarente, 2006). In mammals, there are seven sirtuin homologues (SIRT1 to SIRT7), which possibly have a role in adapting to food deprivation and other environmental stressors. Of these seven, the biological activities of SIRT1 and SIRT2 have been well studied. SIRT1 is a nuclear protein in most cell types. It deacetylates transcription factors and cofactors that regulate several metabolic pathways in energy metabolism (Li *et al*., 2007) and circadian rhythm (Nakahata *et al*., 2008). SIRT1 is believed to have important function in glucose homeostasis and lipid metabolism in various tissues including adipose tissues, liver, pancreas, and skeletal muscle (Kume *et al*., 2010). Other SIRT1 targets are related to stress tolerance, DNA repair, (Nakagawa & Guarente, 2011), and inflammation (Yeung *et al*., 2004). Sir2 mediates transcriptional silencing at selected regions of the yeast genome, and expanded lifespan of yeast mother cells has been shown to be associated with increased the silencing activity of Sir2 at the ribosomal DNA repeats (Kaeberlein *et al*., 1999).

Several reports on association between sirtuins and disease conditions such as diabetes and metabolic diseases; cardiovascular diseases; neurodegenerative diseases; and cancers have been published in recent years; mostly in animal models (Guarente, 2014). Incidentally, all these conditions are associated with aging process. There is substantial evidence to relate sirtuins to the aging process, age-related diseases, and anti-aging interventions. In the present study, an attempt was made to relate sirtuins to a major geriatric syndrome that is frailty. Serum sirtuins (SIRT1, 2 and 3) were significantly lower in frails as compared to nonfrail after adjustment for multiple confounders such as age, gender, diabetes mellitus, hypertension, cognitive impairment, and number of comorbidities. SIRT 1 and 3 levels decreased in patients with diabetes and hypertension, but the levels were even lower in individuals who were frail and had diabetes or hypertension. SIRT 1 and 3 levels also fell as age and the number of comorbidities increased, but the decline was more in frailer subjects compared with nonfrail elderly. The level of SIRT 2 was also lower in frail compared with nonfrail reaching the level of statistical significance.

This the first report associating significantly low circulating sirtuin with frailty. The results of SPR technology was validated by Western blot analysis considered as gold standard for the analysis of proteins. Earlier studies conducted on CHAMP population assessed the SIRT1 expression in SK Hep1 cells grown in the presence of serum samples obtained from frail or nonfrail individuals and found there was no association between frailty and SIRT1 expression in cells. The post hoc analysis suggested that there might be a paradoxical association between low serum-induced SIRT1 expression and robustness (Le Couteur *et al*., 2011). Moreover, the authors of the CHAMP study accepted that their results were unexpected, as high tissue expression of SIRT1 was generally considered to be beneficial, it was induced by CR and expected to be higher in younger animals.

There are some biological parameters which have been used as biomarkers of frailty, namely inflammation (Leng *et al*., 2002, 2004, 2007, 2009), central adiposity, serum albumin, oxidative stress (Wu *et al.,* 2009), vitamin-E (Ble *et al*., 2006), and 25 hydrooxy vitamin-D level (Hirani *et al*., 2013). These parameters are, however, nonspecific and do not indicate any mechanistic pathway. Despite rise in the number of frail patients in clinical practice, so far, no diagnostic biomarker has been identified for frailty, possibly due to decline in multiple physiological functions. Better understanding of the anti-aging effects of CR at a molecular level may ultimately help in the development of potent diagnostic and therapeutic target for frailty, and sirtuins may emerge as key molecules in this regard.

ROC results showed that serum SIRT1 and SIRT3 have great potential to be diagnostic protein marker for frailty for its accuracy in the study cohort. This is the first study to report the clinically diagnostic relevance of SIRT1 and SIRT3 as serum protein marker for frailty.

## Experimental Procedures

### Study groups

Two hundred patients over the age of 60 years visiting Geriatric Medicine Outpatient Department of All India Institute of Medical Sciences, New Delhi, were included in this cross-sectional study. The study was approved by the Institute Ethics Committee (IESC/T-270/01.07.2011), and all participants provided written informed consent. Frailty was diagnosed using Fried's criteria (Fried *et al*., 2001) which is based on the presence of five measurable characteristics: slow motor performance (walking speed), poor endurance and energy (self-report of exhaustion), weakness (grip strength), shrinking (unintentional weight loss), and low level of physical activity. Exclusion criteria included musculoskeletal and neurological diseases that may impair their ability to perform the tasks involved in assessment of frailty. Patients were considered frail if they had three or more of these characteristics and those with two or less were termed nonfrail constituted the control group.

### Estimation of SIRT1, SIRT2, and SIRT3 level in the study group

Two milliliter of venous blood was collected from the patients after assessment and diagnosis. Blood samples were allowed to stand for 30–40 min, and serum separated after 20 min centrifugation was aliquot and stored at −80°C.

#### By surface plasmon resonance

BIAcore 2000 (Pharmacia Biosensor AB, Sweden) was used for all label-free real-time monitoring of target bimolecular interactions. Mouse anti-human SIRT1 monoclonal IgG (Santa Cruz Biotechnology, CA, USA), Goat anti-human SIRT2 monoclonal IgG (Santa Cruz Biotechnology), and Rabbit anti-human SIRT3 polyclonal IgG (Santa Cruz Biotechnology) were immobilized on three different flow cells of CM5 sensor chip separately via amine coupling kit (Pharmacia Biosensor AB). The system was equilibrated with running buffer, that is, HBS-EP buffer (10 mm HEPES pH 7.4, 150 mm sodium chloride, 3 mm EDTA, 0.005% polysorbate 20), and maintained at a flow rate of 5 mL/min. The experimental flow cell dextran was activated using 1:1 volume mixture (110 μL each) of EDC (N-ethyl-N'-3 diethylaminopropylcarbodiimide) (75 μg/μl) and NHS (N-hydroxysuccinimide) (11.5 μg/μL). Antibody was diluted to 100 μg/mL in 10 mm sodium acetate (pH 5) and injected over the activated chip surface. Unreacted groups were blocked by ethanolamine (pH 8.5). Standard graphs were prepared by passing different known concentration of pure SIRT1 (0.62, 3.12, 6.25, 18.75, 31.25, and 62.25 ng/μL), SIRT2 (1.7, 8.5, 17, 25.5, 34, 42.5, 51, 68 ng/μL), and SIRT3 (0.6, 1.2, 6, 9, 12, 15, 18 ng/μL.) over respective flow cells of sensor chip and respective response unit (RU) values obtained (SIRT1, SIRT2, and SIRT3 proteins were cloned, expressed, and purified in bacterial system). Each sample was analyzed in triplicate.

Serum samples were diluted (1:70) with HBS-EP buffer and allowed to run over immobilized antibodies. The concentration of SIRT1, SIRT2, and SIRT3 of study groups was determined from respective standard curves.

#### By Western blot

Serum samples collected from 12 frail and 12 nonfrail individuals were subjected to removal of major interfering proteins using plasma 7 multiple affinity removal spin cartridge according to the manufacturer's protocol (Agilent Technologies, Santa Clara, CA, USA). Total protein concentration was determined using (bicinchoninic acid assay (BCA) using bovine serum albumin as standards. 50 μg of total protein was separated by sodium dodecyl sulfate polyacrylamide gel electrophoresis (SDS–PAGE). After electrophoresis, protein was transferred to polyvinylidenedifluoride (PVDF) membranes (MDI Membrane Technologies, India). The membranes were then blocked in 5% nonfat dry milk prepared in TBS (10 mm Tris pH 7.5, 150 mm NaCl) for 2 h and subsequently incubated with primary antibodies diluted with TBS at 4°C overnight. The following antibodies and titers were used: Mouse anti-human SIRT1 monoclonal IgG (1:300), Goat anti-human SIRT2 monoclonal IgG (1:200), and Rabbit anti-human SIRT3 polyclonal IgG (1:500). After washing with TBS-T (20 mm Tris pH7.5, 500 mm NaCl, 0.05% Tween 20), membranes were incubated with HRP-conjugated secondary antibodies; Goat Anti-Mouse IgG (1:5000; GenScript, Piscataway, NJ, USA), Donkey Anti-Goat IgG (1:1000 dilution; Santa Cruz Biotechnology,), or Goat Anti-Rabbit IgG (1:5000, GenScript) at room temperature for 1 hour. After washing with TBS-T, bands were visualized using enhanced chemiluminescent system (Pierce ECL Western Blotting Substrate; Thermo Scientific, Rockford, IL, USA). Quantification of band intensity was performed using Quantity-one1-D-analysis software (Bio-Rad Laboratories, Hialeah FL, USA).

### Statistical analysis

Analysis was carried out using SPSS Statistics version 17.0 and Stata/IC version 11.1 (Stata Corp LP, College Station, TX, USA). Descriptive analysis was performed for all variables and with percentages or mean (and standard deviation) as appropriate. Baseline comparison between frail and nonfrail population was made using appropriate statistical tests: Student t-test for continuous variables, chi-square test for categorical variables, and ANOVA for comparison of more than 2 categories. ROC curves were constructed to determine best cutoff for SIRT1, SIRT2, and SIRT3. Analysis of covariance was carried out to check the difference in the levels of SIRT1, SIRT2, and SIRT3 between frail and nonfrail after adjusting potential confounders. For statistical significance, *P-*value of < 0.05 was considered as the cutoff.

## Supporting Information

Figure S1 Standard graph generated by passing different concentration of (A) SIRT1 (0.62, 3.12, 6.25, 18.75, 31.25, and 62.25 ng/μL), (B) SIRT2 (1.7, 8.5, 17, 25.5, 34, 42.5, 51, 68 ng/μL), and (C) SIRT3 (0.6, 1.2, 6, 9, 12, 15, 18 ng/μL.) over respective immobilized antibody. The graph was plotted between concentration and the RU value obtained.

Figure S2 Western blot and density analysis showing the linearity of the sensitivity and specificity of different antibodies 1,3,9,12,15,18, and 21 μg of pure SIRT1 (A), SIRT2 (B), and SIRT3 (C) loaded in lane 1–7, respectively.
