# Supplementary figures and images for: Identification of serum sirtuins as novel noninvasive protein markers for frailty
Source: Aging Cell. 2014 Aug 7;13(6):975–80. doi: 10.1111/acel.12260 (PMC4326933; doi:10.1111/acel.12260)

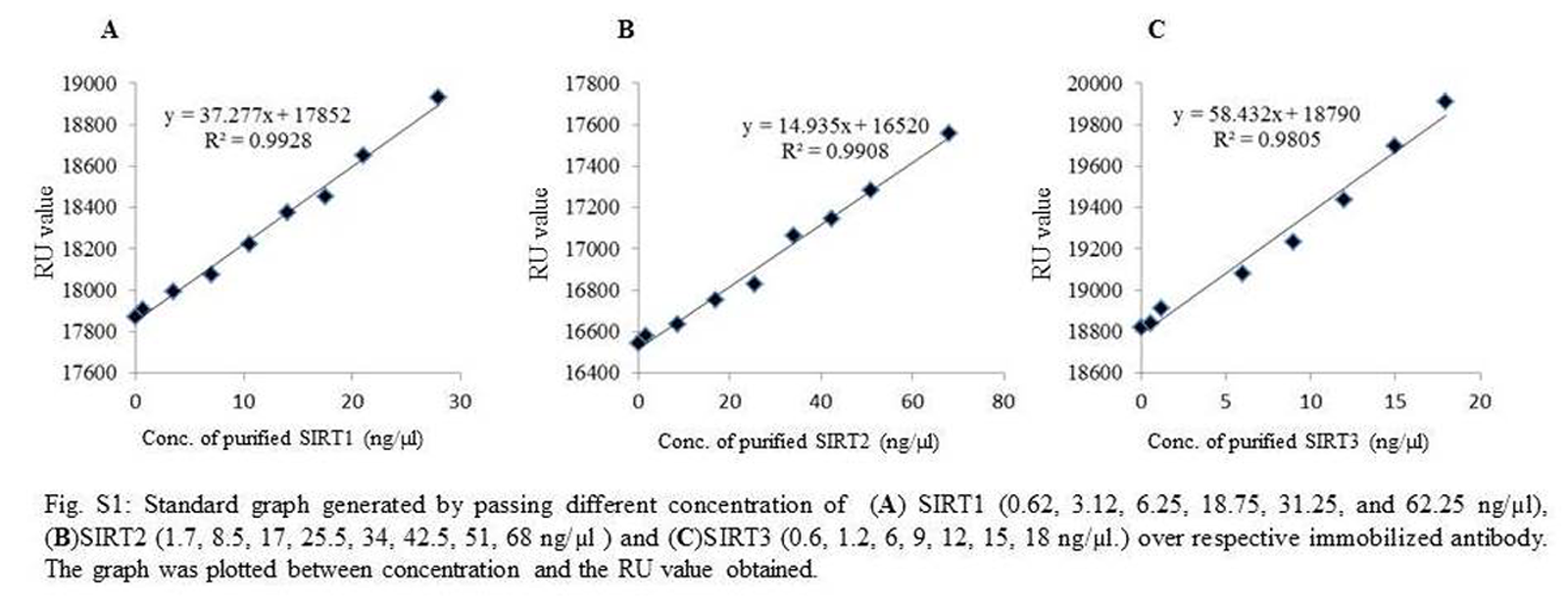

Supplement: Figure S1 — Standard graph generated by passing different concentration of (A) SIRT1 (0.62, 3.12, 6.25, 18.75, 31.25, and 62.25 ng/μL), (B) SIRT2 (1.7, 8.5, 17, 25.5, 34, 42.5, 51, 68 ng/μL), and (C) SIRT3 (0.6, 1.2, 6, 9, 12, 15, 18 ng/μL.) over respective immobilized antibody. The graph was plotted between concentration and the RU value obtained. [file acel0013-0975-sd1.tif]

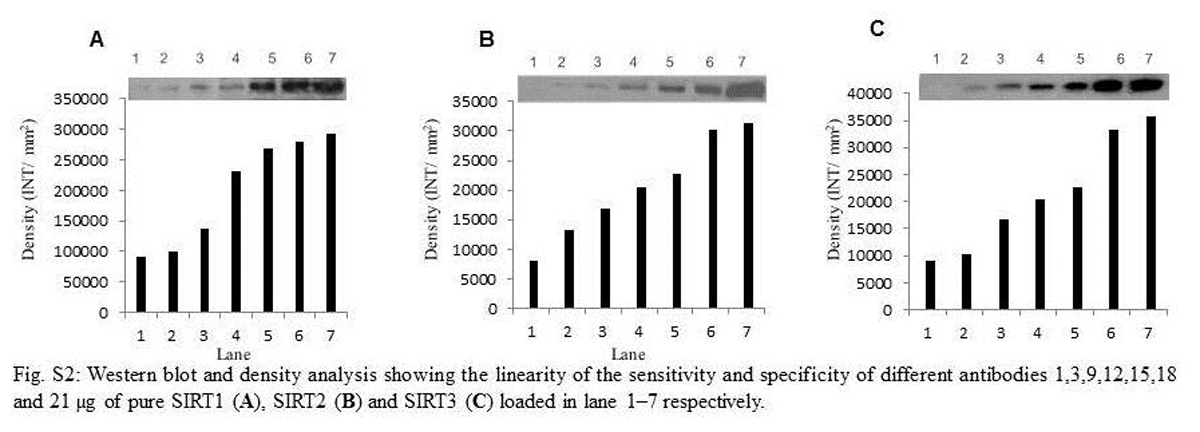

Supplement: Figure S2 — Western blot and density analysis showing the linearity of the sensitivity and specificity of different antibodies 1,3,9,12,15,18, and 21 μg of pure SIRT1 (A), SIRT2 (B), and SIRT3 (C) loaded in lane 1–7, respectively. [file acel0013-0975-sd2.tif]
